# Results of Open and Endovascular Abdominal Aortic Aneurysm Repair According to the E-PASS Score

**DOI:** 10.5935/1678-9741.20160006

**Published:** 2016

**Authors:** Fábio Hüsemann Menezes, Bárbara Ferrarezi, Moisés Amâncio de Souza, Susyanne Lavor Cosme, Giovani José Dal Poggetto Molinari

**Affiliations:** 1 Faculdade de Ciências Médicas da Universidade Estadual de Campinas (FCM-UNICAMP), Campinas, SP, Brazil.; 2 Vascular Surgery Department, Hospital das Clinicas da Faculdade de Ciências Médicas da Universidade Estadual de Campinas (HCFCM-UNICAMP), Campinas, SP, Brazil.

**Keywords:** Postoperative complications, Surgical Procedures, Operative, Aortic aneurysm, abdominal, Endovascular Procedures

## Abstract

**Introduction:**

Endovascular repair (EVAR) of abdominal aortic aneurysm has become the standard of care due to a lower 30-day mortality, a lower morbidity, shorter hospital stay and a quicker recovery. The role of open repair (OR) and to whom this type of operation should be offered is subject to discussion.

**Objective:**

To present a single center experience on the repair of abdominal aortic aneurysm, comparing the results of open and endovascular repairs.

**Methods:**

Retrospective cross-sectional observational study including 286 patients submitted to OR and 91 patients submitted to EVAR. The mean follow-up for the OR group was 66 months and for the EVAR group was 39 months.

**Results:**

The overall mortality was 11.89% for OR and 7.69% for EVAR (*P*=0.263), EVAR presented a death relative risk of 0.647. It was also found a lower intraoperative bleeding for EVAR (OR=1417.48±1180.42 mL *versus* EVAR=597.80±488.81 mL, *P*<0.0002) and a shorter operative time for endovascular repair (OR=4.40±1.08 hours *versus* EVAR=3.58±1.26 hours, *P*<0.003). The postoperative complications presented no statistical difference between groups (OR=29.03% *versus* EVAR=25.27%, *P*=0.35).

**Conclusion:**

EVAR presents a better short term outcome than OR in all classes of physiologic risk. In order to train future vascular surgeons on OR, only young and healthy patients, who carry a very low risk of adverse events, should be selected, aiming at the long term durability of the procedure.

**Table:** 

**Abbreviations, acronyms & symbols**	
AAA	= Abdominal aortic aneurysms
ASA	= American Anesthesiology Society risk classification
E-PASS	= Estimation of Physiologic and Surgical Stress
EVAR	= Endovascular repair
OR	= Open repair
PRS	= Physiologic Risk Score
ROC	= Receiver operating characteristic

## INTRODUCTION

Recently, Silva^[1]^ published an editorial reflecting on the cost/benefit and technical aspects in order to choose the best option between open (OR) and endovascular (EVAR) procedures in the repair of abdominal aortic aneurysms (AAA). Case series and randomized clinical trials comparing the results between OR and EVAR demonstrate that there is a reduction in the 30-day mortality associated with the less invasive technique^[2-6]^. Firwana et al.^[7]^ reviewed six randomized clinical trials and concluded that the risk of death from the procedure is reduced to one third (RR 0.35 95% CI 0.19-0.64) using the endovascular technique. In the most recent Cochrane Library review^[8]^ the same results were found with a relative risk of death of 0.33 (95% CI 0.20-0.55) using the endovascular technique. Nonetheless, OR presents good long-term results and lower incidence of reinterventions^[9-13]^. The objective of this paper is to review the morbidity and mortality associated with the repair of the AAA in two series of patients submitted to OR, and EVAR, in a public university hospital. For a better evaluation of the clinical benefit, the patients were classified according to the physiologic risk component of the E-PASS (Estimation of Physiologic and Surgical Stress)^[9]^ score. Based on the results, the authors present their opinion regarding the actual indications for OR.

## METHODS

This is a retrospective cross-sectional observational study with data extracted from the patients' hospital charts submitted to AAA repair in a public university hospital located in the countryside of the State of Sao Paulo. The patients were submitted to open AAA repair (OR) from February 2000 to September 2013 and to endovascular AAA repair (EVAR) from June 2005 to June 2013, when this technique was introduced into the hospital practice. Patients with a diagnosis of ruptured abdominal aneurysm or inflammatory abdominal aneurysms were excluded from the study because they cannot be evaluated by the adopted risk score. All patients whose hospital charts data were not complete were also excluded from the study, resulting in the inclusion of a total of 286 patients submitted to OR and 91 patients submitted to EVAR. The mean time of follow-up for the OR group was 66 months and for the EVAR group was 39 months.

Table 1 presents the demographic data of the study groups. A significant difference was found between groups regarding age (68.3 years old in the OR group *versus* 73.8 years old in the EVAR group, *P*<0.0001), pulmonary risk (8.74% risk present in the OR group *versus* 47.25% risk present in the EVAR group, *P*<0.001), and presence of renal disease (17.55% risk present in the OR group *versus* 30.77% risk present in the EVAR group, *P*=0.0035). Patients submitted to EVAR also had a higher American Anesthesiology Society (ASA) risk classification (16.78% of patients with ASA 4 in the OR group *versus* 40.66% of patients with ASA 4 in the EVAR group, *P*<0.0001).

**Table 1:** Demographic data of the operated groups (2000-2013).

	Open Repair		Endovascular Repair		P-value
Variable	n = 286		n = 91		
Age	68.31±8.19	years	73.83±8.68	years	<0.0001
Male Gender	83.92	%	84.62	%	0.87
White Race	90.91	%	91.21	%	0.93
Arterial Hypertension	75.44	%	84.62	%	0.07
Smoking	88.07	%	90.11	%	0.59
Cardiac Disease	6.29	%	5.49	%	0.78
Lung Disease	8.74	%	47.25	%	<0.0001
Diabetes	13.29	%	12.09	%	0.76
Renal Disease	17.55	%	30.77	%	0.0035
ASA 4	16.78	%	40.66	%	<0.0001
PSI 3+4	6.94	%	7.69	%	0.14

Smoking=active smoking or past smoking history; cardiac disease=presence of disease in category 3 or above classification of the Society for Vascular Surgery (SVS)^[14]^; lung disease=presence of disease in category 2 or above of the SVS; renal disease=presence of a creatinine level above 1.5 mg/dL; ASA=risk classification according to the American Anesthesiology Society; PSI=Performance Status Index.measures the level of physical activity of the patient used according to the E-PASS score^[9]^

**Fig. 1:**
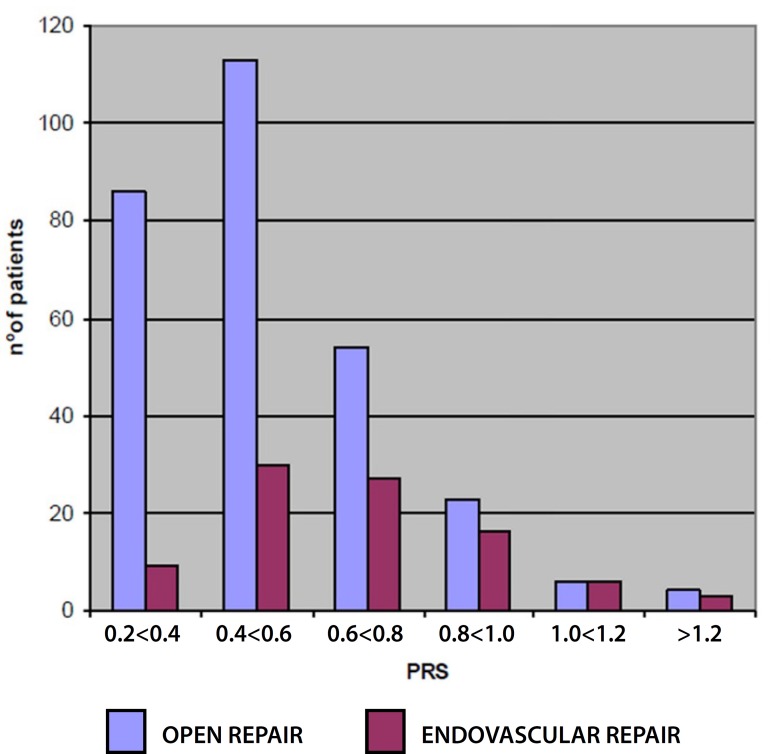
Distribution of patients according to surgical technique and physiologic risk score (PRS).

The physiologic risk classification (Physiologic Risk Score - PRS) was done by adopting the same criteria previously published by Menezes and Souza^[9]^, using the E-PASS score originally published by Haga et al.^[15]^. This risk score varies from zero to a value of 1.2. Higher values correspond to a higher risk of postoperative complications. For OR, the value of 0.4 is considered low risk. Figure 1 presents the distribution of patients according to surgical technique and PRS classification. There was a significant difference between the two groups, with the EVAR group presenting a higher risk (mean PRS 0.54±0.21 for OR *versus* 0.69±0.25 for EVAR, *P*<0.0004).

Surgical morbidity was evaluated based on the classification proposed by Tang et al.^[16]^. According to their classification, zero represents no postoperative complications. The value of one represents a minor complication limited to the incision or that does not need medical intervention. The value of two represents complications that require medical intervention but does not need artificial support. The value of three represents complications that require artificial support to maintain vital organ function (lung, kidney or cardiac). The value of four represents the in-hospital death of the patient, even if it happens after the 30^th^ postoperative day. The duration of the surgical procedure, the intraoperative blood loss and the length of the hospital stay were also tabulated.

All data was inserted into a data bank (Microsoft Accesss 2003) and submitted to statistical analysis by the Institution's Statistical Support Group. The exploratory data was presented as frequency, percentage, mean, standard deviation, minimum and maximum values. The comparison between groups for the numeric data was performed with the Mann-Whitney test and for the categorical data the Qui-Square or the Exact Test of Fisher were used. Sensibility and specificity of the PRS were evaluated with Receiver Operating Characteristic (ROC) curves. A 5% significance level was adopted. This work was approved by the Institution's Ethics Committee on July 23^rd^ 2013, receiving the identification number 343.087.

## RESULTS

Table 2 presents the results of the intraoperative blood loss, surgical procedure and hospital stay duration, PRS value, global mortality (category 4), major complications (category 2) and the need of artificial mechanical support (category 3) for the OR and EVAR groups, all values achieved statistical significance, when compared between groups.

**Table 2:** Surgical result of OR and EVAR.

Variable	Open Repair		Endovascular Repair		P-value
Bleeding	1417.48 ±1180.42	mL (mean±sd)	597.80±488.81	mL (mean±sd)	< 0.0002
Operative Length	4.40 ±1.08	hours (mean±sd)	3.58±1.26	hours (mean±sd)	< 0.0003
PRS	0.54±0.21		0.69±0.25	(mean±sd)	<0.0004
Hospital Stay	8.68±10.56	days (mean±sd)	9.37±10.65	days (mean±sd)	0.0244
Mortality	11.89	%	7.69	%	0.263
Major Complications	24.83	%	25.27	%	0.35
Mechanical Support	4.2	%	0		Not calculated

PRS=physiologic component of the E-PASS score; major complications=complications that required medical intervention, corresponding to category 2 of Tang et al.^[16]^; mechanical support=complications that required the use of artificial mechanical support, corresponding to category 3 of Tang et al.^[16]^

EVAR presented a smaller intraoperative blood loss (597.8 mL for EVAR *versus* 1,417.5 mL for OR, *P*<0.001), a shorter operation time (3.6 hours for EVAR *versus* 4.4 hours for OR, *P*<0.001) and a higher physiologic risk classification (0.69 EVAR *versus* 0.54 OR, *P*<0.001). Hospitalization time could be considered equal for the two groups, (9.4±10.7 days for the EVAR group *versus* 8.7±10.6 days for the OR group) even though there was statistical difference among them (*P*=0.0244).

Global mortality was 11.89% for the OR and 7.69% for EVAR (*P*=0.263). Even though there was no statistical difference, the mortality of the EVAR group was 35.3% lower than the mortality of the OR group (RR=0.647). It is important to note that the EVAR group presented a higher percentage of patients in the higher physiologic classification risk (30% in the OR group *versus* 57% in the EVAR group). There was no statistical difference between the groups regarding complications that required medical intervention (value of 2 according to Tang et al.^[16]^. All patients submitted to EVAR that required artificial support in this casuistry died, resulting in no patients in this category (value of 3 according to Tang et al.^[16]^). In the OR group 12 (4.2%) patients were in this category.

Table 3 presents the results of morbidity according to the classification of the physiologic risk of the patients, comparing the OR and EVAR groups. For the OR group 69.6% of the patients were included in low risk groups 0.2 to 0.6 of PRS, and only 42.6% of the patients submitted to EVAR were in these risk groups.

**Table 3:** Distribution of patients submitted to OR or EVAR into the morbidity classification, according to the PRS value. Morbidity Groups - Open Repair

PRS	n	0	%	1	%	2	%	3	%	4	%
0.2<0.4	86	62	72.09	4	4.65	14	16.28	3	3.49	3	3.49
0.4<0.6	113	76	67.26	4	3.54	22	19.47	4	3.54	7	6.19
0.6<0.8	54	29	53.70	1	1.85	12	22.22	3	5.56	9	16.67
0.8<1.0	23	3	13.04	1	4.35	9	39.13	2	8.70	8	34.78
1.0<1.2	6	1	16.67	0	0	1	16.67	0	0	4	66.67
>1.2	4	0	0	0	0	1	25.00	0	0	3	75.00
Morbidity Groups - Endovascular Repair											
PRS	n	0	%	1	%	2	%	3	%	4	%
0.2<0.4	9	5	55.56	3	33.33	1	11.11	0	0	0	0
0.4<0.6	30	11	36.67	11	36.67	7	23.33	0	0	1	3.33
0.6<0.8	27	16	59.26	2	7.41	6	22.22	0	0	3	11.11
0.8<1.0	16	5	31.25	1	6.25	7	43.75	0	0	3	18.75
1.0<1.2	6	3	50.00	3	50.00	0	0	0	0	0	0
>1.2	3	1	33.33	0	0	2	66.67	0	0	0	0

Value zero represents no postoperative complication, value one represents a minor complication limited to the incision or that did not need medical intervention, value two represents complications that required medical intervention but did not need artificial support, value three represents complications that required artificial support to maintain vital organ function (lung. kidney or heart), value four represents the in-hospital death of the patient, even if it happened after the 30^th^ postoperative day^[16]^.

**Fig. 2:**
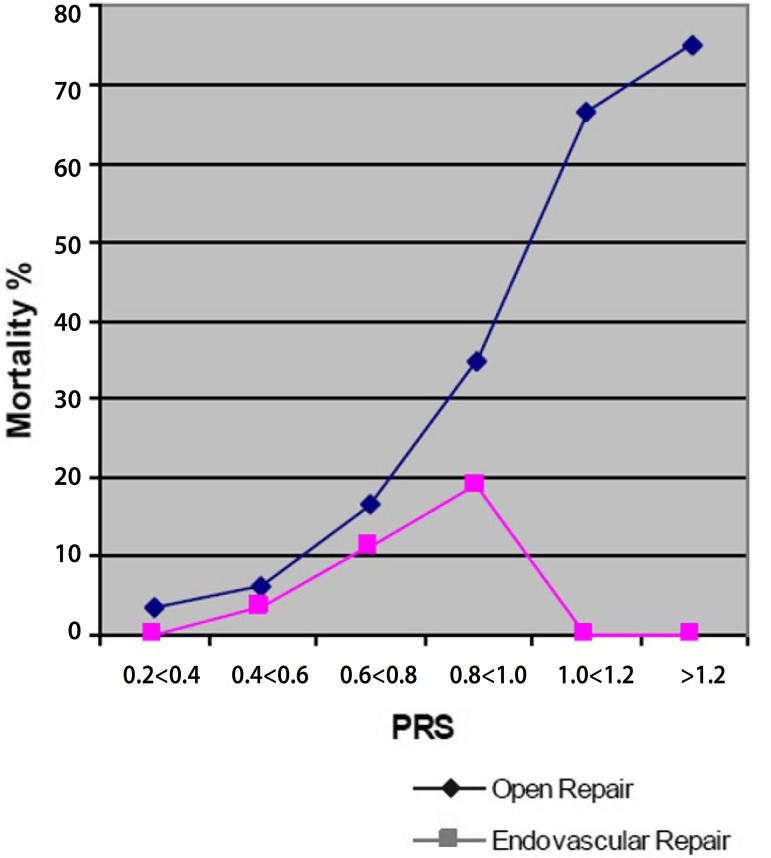
Surgical mortality in the OR and EVAR groups according to PRS.

Figure 2 presents the results of surgical mortality according to physiologic risk (PRS). In the lower surgical risk group (PRS<0.6) the mortality in the EVAR group was 46% of the mortality in the OR group. As the physiologic risk increases, there is an exponential elevation of mortality in the OR group. In the EVAR group an elevation of mortality also occurs, but it is kept between 33% and 46% of the mortality in the OR group. There was no mortality in the EVAR group for the patients with a very high physiologic risk (PRS > or = to 1), where the surgical mortality in the OR group is approximately 70%.

**Fig. 3:**
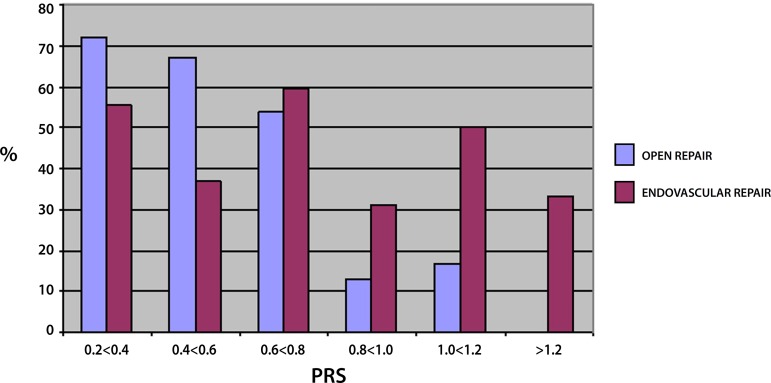
Percentage of patients that did not present postoperative complications in the OR and EVAR groups, according to PRS.

**Fig. 4:**
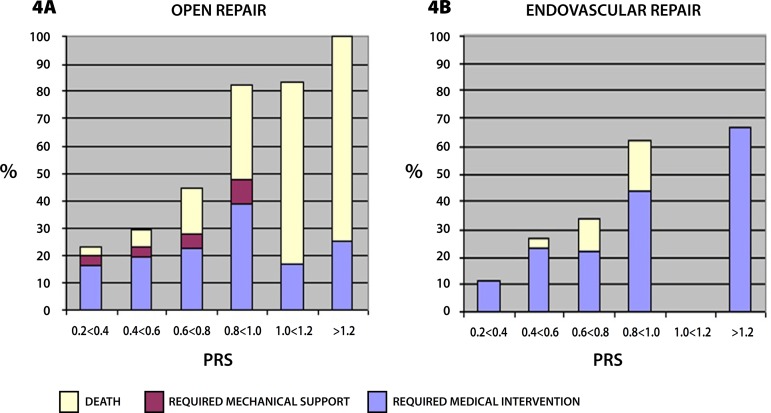
Percentage of patients that presented major postoperative complications or death in OR and EVAR groups, according to PRS.

Figures 3 and 4 demonstrate respectively the percentage of patients that did not present a surgical complication and the percentage of patients that presented complications that required medical intervention and artificial mechanical support. As the physiologic risk increases the percentage of patients in the OR group that does not present any complication decreases proportionally, which is not seen in the EVAR group, probably related to the low seriousness type of complication found in the EVAR group, such as access site hematomas. Both groups presented an increase in complications that required medical intervention as the physiologic risk became greater, demonstrating that both groups represent patients that carry important co-morbidities besides the AAA. Figure 4 shows a lower complication rate of the OR group at the higher physiologic scores because most of the patients did not survive the procedure.

**Fig. 5:**
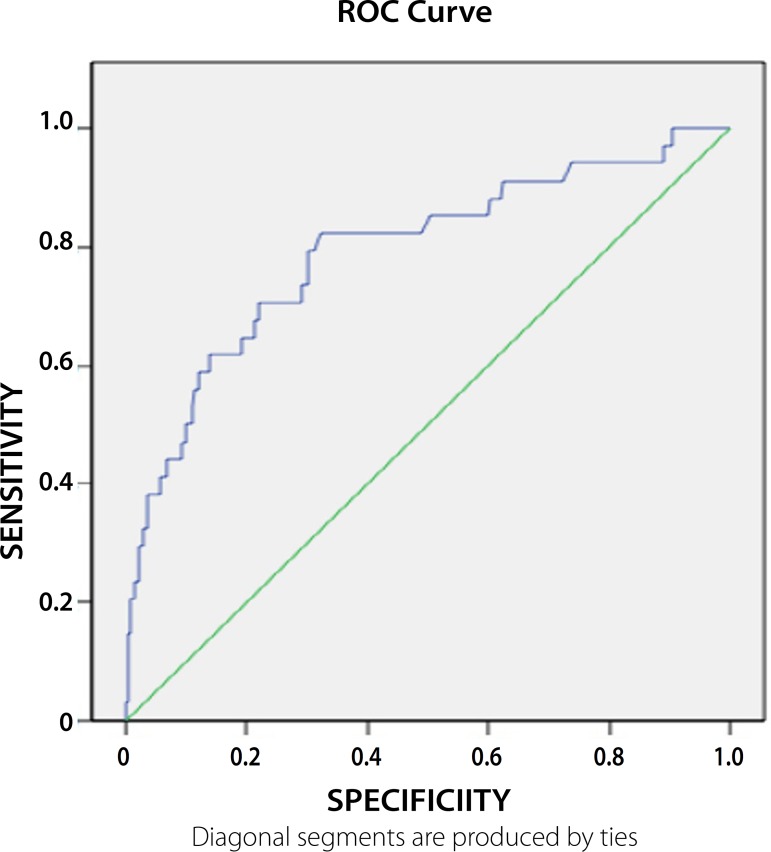
ROC curve for PRS versus survival in OR.

**Fig. 6:**
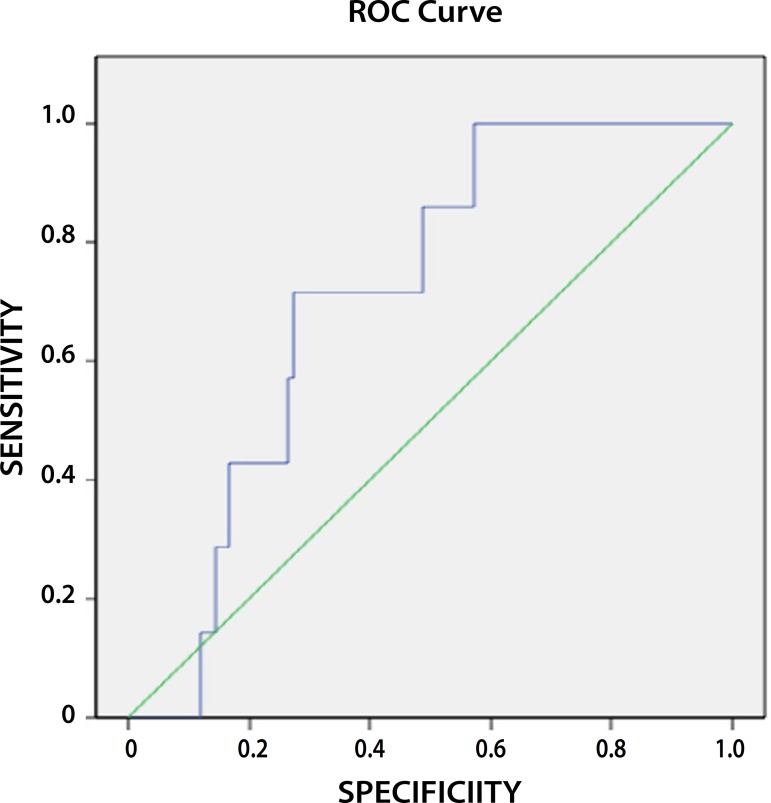
ROC curve for PRS versus survival in EVAR.

Mortality ROC curve (Table 4 and Figures 5 and 6) were generated for the PRS values in order to establish a value with a higher probability of discriminating patients that would not survive the AAA repair if submitted to either technique. The cut value for the EVAR group was higher (0.754 for the EVAR group *versus* 0.631 for the OR group), demonstrating a less invasive nature of EVAR.

**Table 4:** ROC curve discriminating values of PRS *versus* survival.

Repair	AUC	Std. Error	P-value	95% CI		Cut value	sensibility	1-specificity
				Lower Bound	Upper Bound			
EVAR	0.711	0.073	0.065	0.568	0.853	0.754	0.73	0.30
						0.600	0.86	0.56
OR	0.792	0.046	0.000	0.703	0.882	0.631	0.71	0.44
						0.400	0.93	0.72

## DISCUSSION

The E-PASS score was chosen because it is simple and easy to use, when compared to other risk scores, and presents a good capacity to anticipate the mortality of open AAA repair, as previously demonstrated^[9,15-20]^.

Compared to other studies, a higher than expected mortality for the OR and EVAR groups was found in the present study. Goshima et al.^[21]^ state that the standard result for OR should be 3.1% and in their study, the EVAR mortality was null. But the same authors, when presenting the complex cases, relate a hospital mortality of 14.1%. Jackson et al.^[22]^ have found in the Medicare population a mortality of 3.13% for the OR group and 0.7% for the EVAR group; a result that is very similar to the subgroup of very low physiologic risk in this study, in which the mortality of the OR group was 3.49% and absent in the EVAR group. Egorova et al.^[23]^, also presenting results related to the Medicare population, show that a small group of patients, with high surgical risk factors, such as congestive heart failure and advanced renal insufficiency, presents a mortality as high as 11%, which is also compatible with our results for the group with a higher physiologic risk score. In the EVAR-2 study^[24,25]^, patients unsuitable for open repair were randomized to EVAR or clinical follow-up, the 30-day mortality of the operated group was 9% (95% CI 5-15%). The above literature reinforces the concept that the surgical result, even in the EVAR cases, is dependent on the preoperative physiologic status of the patient, as it is clearly seen in this study. Since a large proportion of patients in this cohort was considered high risk, this could have contributed to the unexpected higher mortality rate in the EVAR group.

Another factor to be considered is the variation in surgical result dependent on the anatomical configuration of the aorta and access arteries. A shorter, tortuous, dilated proximal neck has a negative influence on the results, as tortuous and narrow iliac vessels also do. In the present study, these data were not collected and therefore it is not possible to reach a conclusion and it may be subject of a future study.

A third factor to be considered in relation to a higher mortality is the learning curve of the surgical staff. Cohnert et al.^[26]^ when describing their initial experience with EVAR presented a 30-day mortality of 18.9%, while having a 10.9% mortality in the OR group operated in the same time frame. Our hospital is a tertiary teaching center, with intense participation of training residents in the operations, for whom the learning curve is always in the beginning, since the group renovates every year. Even though under strict supervision of the hospital teaching staff, this may certainly have an influence on the final results, specially because this learning curve is also seen in the anesthesia performance, as well as on the postoperative intensive care unit performance.

It can be seen as a rule in the literature, that the mortality of the EVAR technique is one third the mortality of the OR technique. Recently it was published that the risk of any adverse events during EVAR is 42% less than for OR^[27]^. This study has observed that during the time frame of 2003 and 2010 the global mortality of AAA repair (including EVAR and OR) fell from 7.4% to 4.4%. In the same period the percentage of patients receiving EVAR increased from 41.1% to 75.3%. In the present study the EVAR mortality was approximately half of the OR mortality, which is within the upper limits of the 95% CI of the decrease in mortality described for EVAR in most series (0.55-0.64). Nonetheless, this gain represents a significant improvement in surgical mortality, especially in the higher physiologic risk patients. In the Brazilian literature, Saadi et al.^[28]^ presented very good results in their initial experience with EVAR with no mortality in 25 patients operated for AAA, while Mendonça et al.^[29]^ found a operative mortality of 5.45% for OR and 6.55% for EVAR.

There were several risk scores proposed for EVAR in order to forecast postoperative complications^[30-32]^. In these studies the authors agree that even though the nature of the procedure is less invasive, the physiologic risk of the patients play an important role in the final results, besides the above mentioned anatomical factors.

The Society for Vascular Surgery^[33]^ proposes that AAA patients with a good operative risk should be submitted to OR, seeking a durable procedure. In order for this to be true the operative mortality of OR should be equivalent to EVAR. This can not be expected if all AAA patients, encompassing all classes of physiological risk, are seen as a single group. In this study, it was found that for patients with a low physiologic risk score (PRS < or=0.6) the operative mortality is equal to the recommended international standards, which is lower than a 6% mortality. Even for these low risk patients, EVAR presented a better result and may justify the use of this technique as first choice, if patients are conscious of the necessity of a rigorous follow up to identify and treat future complications^[8,10,11]^. For the subgroup of patients with a higher physiologic risk score, the 30-day OR operative mortality increases exponentially with the risk, rendering EVAR the only choice. In much selected high risk cases, only clinical observation may be the most appropriate choice.

This study corroborates the findings of a shorter operative time, lesser bleeding and a smaller incidence of severe adverse events that required artificial support of vital organ function in the EVAR group, as seen in Table 2.

The authors acknowledge the weakness of a retrospective study, because of the expected deficiencies of gathering information from hospital charts. Nonetheless, the objectiveness of the collected data makes it trustable information, which is usually correctly annotated in the files validating the results presented.

This local data analysis may represent the clinical picture found in the public university hospitals of Southeast Brazil, which are focused in offering government financed health services to a low socio-economical population and serve as the main training centers of future peripheral vascular surgeons. It may also contribute to a more solid decision on which is the best operative technique, and how to continually improve the surgical results.

One of the main concerns today of the teaching hospitals, all over the world, is how to teach the OR technique to future generations of vascular surgeons^[34-36]^; since EVAR presents a series of advantages that restrict the indications of OR to a few cases of complex anatomy, which usually do not present a low operative risk, and for whom the training physician has a low chance of performing as the main surgeon. One area for future research is how to implement simulated OR for training, increasing the exposure of the young surgeons to open procedures^[35]^.

Another important concern is the bias created when a young surgeon needs to decide on which technique to offer, taking into account that they have been exposed exclusively to EVAR during their formative years^[37-39]^. The economical aspect should also be considered because of the higher costs associated with EVAR. Another hindrance could be the pre-acquired concepts on modern surgical techniques that patients bring from the electronic media, which generates a layperson preference^[1,40,41]^, and also the manner both techniques are offered to patients by the attending physician^[42]^.

## CONCLUSION

As reported by the present study, the short term results of EVAR are superior to OR in all classes of physiologic risk. When selecting patients for the training of new vascular surgeons on OR, teaching hospitals should carefully select young and healthy patients, who carry a favorable anatomy and a very low risk of postoperative adverse events, considering the fact that these patients could benefit from the good long-term durability of OR.

**Table:** 

**Authors' roles & responsibilities**	
FHM	Study design; implementation of projects and/or experiments; analysis and/or interpretation of data; statistical analysis; manuscript writing or critical review of its contents; final approval of the manuscript
BF	Analysis and/or interpretation of data; final approval of the manuscript
MAS	Analysis and/or interpretation of data; final approval of the manuscript
SLC	Analysis and/or interpretation of data; final approval of the manuscript
GJDPM	Analysis and/or interpretation of data; manuscript writing or critical review of its contents; final approval of the manuscript
